# Threat Assessment and Risk Analysis (TARA) for Interoperable Medical Devices in the Operating Room Inspired by the Automotive Industry

**DOI:** 10.3390/healthcare11060872

**Published:** 2023-03-16

**Authors:** Andreas Puder, Jacqueline Henle, Eric Sax

**Affiliations:** 1 Embedded Systems, Getinge AB, 76437 Rastatt, Germany; andreas.puder@getinge.com; 2 Embedded Systems and Sensors Engineering (ESS), FZI Research Center for Information Technology, 10117 Berlin, Germany; henle@fzi.de; 3 Institute for Information Processing Technologies (ITIV), Karlsruhe Institute of Technology (KIT), 76131 Karlsruhe, Germany

**Keywords:** safety, security, medical devices, automotive, Failure-Mode and Effect Analysis (FMEA), Threat Assessment and Risk Analysis (TARA), processes

## Abstract

Prevailing trends in the automotive and medical device industry, such as life cycle overarching configurability, connectivity, and automation, require an adaption of development processes, especially regarding the security and safety thereof. The changing requirements imply that interfaces are more exposed to the outside world, making them more vulnerable to cyberattacks or data leaks. Consequently, not only do development processes need to be revised but also cybersecurity countermeasures and a focus on safety, as well as privacy, have become vital. While vehicles are especially exposed to cybersecurity and safety risks, the medical devices industry faces similar issues. In the automotive industry, proposals and draft regulations exist for security-related risk assessment processes. The medical device industry, which has less experience in these topics and is more heterogeneous, may benefit from drawing inspiration from these efforts. We examined and compared current standards, processes, and methods in both the automotive and medical industries. Based on the requirements regarding safety and security for risk analysis in the medical device industry, we propose the adoption of methods already established in the automotive industry. Furthermore, we present an example based on an interoperable Operating Room table (OR table).

## 1. Introduction

Today, hospitals are increasingly equipped with Internet of Things (IoT) devices, but are not entirely aware of the security and privacy implications thereof [1]. Although a hospital may be certified for Health Insurance Portability and Accountability Act (HIPAA), which is a 1996 U.S. law that governs the security and privacy of Protected Health Information (PHI) and patient access to their medical records [2], they are not prepared for a shared network of IoT and other medical devices [1]. In addition, prior risk management for medical devices mainly addressed functional safety and therefore did not include cybersecurity [3]. Cybersecurity in the healthcare industry, including hospitals, is a relatively new topic [4] since it has been slow to prioritize cybersecurity and is lagging behind other industries in protecting their systems and patient data. To address this issue, hospitals must allocate significant resources toward improving their cybersecurity defenses [5].

A ransomware attack that first increased public awareness of cybersecurity issues in hospital environments happened in 2016 in the Hollywood Presbyterian hospital [6], followed by further ransomware attacks. During the COVID-19 pandemic, these attacks have continued to increase [7], and COVID-19 was the predominant lure in attacks via e-mail [8]. In 2020, a patient had to be transported to another hospital due to a ransomware attack on a German hospital. Even though it could not be entirely proven that this delay caused the patient’s death, this incident represents the first case where the ransomware attack was suspected of having led to a patient’s death [9].

As cybersecurity threats and risks evolve, so do their countermeasures; still, no device can be fully protected [10]. Furthermore, several agencies also see the need to take action in the medical device industry. Accordingly, standards, guidelines, and regulations have been published that deal with the potential harm and life cycle risks from cybersecurity incidents [3]. Thus, threat modeling is recommended by several of these (Section 2).

Cybersecurity risks are also a serious issue in the context of software-dominated Electric/Electronic architectures (E/E architectures) in the automotive industry. In 2010, a security analysis exposed a way of attacking vehicle Electronic Control Units (ECUs) with the goal of embedding malicious software [11]. Furthermore, in 2015, a hacker demonstrated how to remotely start a vehicle engine by attacking a connected mobile app [12].

Alongside the rising importance of Vehicle-to-Everything Communication (V2X) and updatable Service-Oriented Architectures (SOAs), the development of secure E/E architectures and data privacy has become increasingly significant. Furthermore, the goal of developing highly automated vehicles uncovers the growing significance of functional safety being guaranteed during the whole product life cycle. Therefore, standards and regulations, as well as methods, were published to enable the assessment of safety, security, and privacy-compliant development processes. With the goal of measuring the fulfilment of these requirements, models were developed. However, while the trends regarding connectivity, Software Over The Air (SOTA) updates, and automation are not yet established in the industry, these methods need to be adapted and enhanced constantly. Furthermore, vehicles have a growing number of internal and external interfaces that enable connectivity and communication with other devices or infrastructure. Alongside these developments, the importance of security rises constantly. Risks and security attacks have been consistently demonstrated over at least the past 15 years [13].

Ensuring quality in the face of risks and threats is a mandatory requirement for businesses in healthcare. According to a 2017 study by McKinsey [14], the direct costs associated with poor quality worldwide in the medical device industry in 2016 were estimated to be between USD 18 billion and USD 22 billion. These costs included the labor required for remediation efforts, internal and external quality failures, and non-routine external failures. The study also found that the direct costs of poor quality accounted for a significant portion of total sales in the medical device industry, with estimates ranging from 11.6% to 16.3% of every sale’s USD spent on these costs in 2016. Thus, improving quality throughout the life cycle of a medical device by implementing effective processes and methods can have a positive economic impact. In addition to traditional concerns around quality and safety, the growing importance of cybersecurity in the medical device industry means that companies must also prioritize quality in this area to ensure the security and protection of patient data.

Problem: As more and more devices in the Operating Room (OR) are connected with each other and are becoming part of the IoT, devices and networks in hospitals need to be secured against potential attackers. There is little knowledge about the security threats in the OR today and most manufacturers still rely on traditional security measures, such as *security by obscurity* [15] or *Defense-in-Depth* strategies [16]. While the first is already proven as a non-efficient measure, the latter is still a prevalent strategy, although it is considered outdated and is being increasingly replaced by a zero-trust security model in other fields [17]. In addition, the responsibility for hospital security is not clear, as it is shared among device manufacturers, healthcare providers, security experts, patients, and governing bodies [18].

Cybersecurity is still in the process of being recognized as vital in the whole healthcare industry (Section 1). This is reflected by the numerous collections of standards and guidelines that exist around the world, which are constantly being renewed or reworked (Section 2.5). As a result, there are not yet sufficient processes and methods in place that are comparable to those in other industries, such as the automotive sector. Additionally, cybersecurity must be approached differently in different sectors of the healthcare industry. The growing share of software in the healthcare sector has to be distinguished. While, on the one hand, smartphone apps for healthcare make it easier for patients to communicate with their attending physician and collect health data, medical devices such as surgical robots operate in a different environment. Therefore, these fields face different threats and risks and need to be regarded in other ways.

Contribution: In [19], we showed that the main trends in both the automotive and medical device industries face challenges such as higher connectivity, SOA, and SOTA updates. We presented a mixed E/E architecture for OR tables in order to face the challenges of future medical devices and also addressed security risks by introducing Identity and Access Management (IAM). Following up on this, we investigated automotive security processes and methods for improving the security of connected medical devices. By using the existing threat models in the automotive industry, we evaluated their suitability for exposing security risks and examined their relation to safety.

Furthermore, we examined the threat modeling recommended and required by different standards and guidelines in the medical context. Here, risk evaluation is important and already well-established, but the industry is just starting to adopt methodological approaches for security risk analysis. We focused on models originating from the automotive industry that are applicable to medical devices. Therefore, an OR table represents an appropriate representative for the execution of a Threat Analysis and Risk Assessment (TARA) in order to identify the threat landscape for OR equipment.

Outline: In this article paper, we first provide the background and state of the art on the topic of security and safety in development processes and research regarding medical device communication in ORs. We then compare and contrast safety and security standards, guidelines, and methods in these fields (Section 2). In Section 3, we review related work in the automotive and medical fields. Based on the overview of security threat models from Section 2, we analyze necessary adoptions to the medical context (Section 4). Furthermore, we present a TARA for an interoperable OR table in an OR network and combine it with risk analysis approaches (Section 5). Lastly, we summarize our work and provide an outlook on future directions for research in this area (Section 6).

## 2. Background and State of the Art

### 2.1. Medical Communication Systems in the Operating Room

The communication of medical devices in the past few decades has been dominated by proprietary communication protocols that have been bilaterally developed by medical device manufacturers [19]. In terms of security, they often relied on countermeasures such as *security by obscurity*, which is considered as insecure today (Section 1), or limiting the invocable functionality via the network interfaces [20]. However, in particular, robotic medical devices in the OR will need to be competitive in the future regarding their connectivity interface [21].

These developments led to several projects with the aim of introducing Cyber Physical Systems (CPSs) and improving manufacturer-independent interoperability in the OR. The Smart Cyber Operating Theater^®^ (SCOT^®^) project, started by the Tokyo Women’s Medical University, focuses on the use of CPSs in the Hybrid Operating Room (HOR) [22], which allows imaging procedures to be performed during surgery in a single OR. The Medical Device Plug and Play (MDPnP) project aims to enable the use of heterogeneous medical devices from different manufacturers in a medical device system and has introduced the concept of the Integrated Clinical Environment (ICE) to describe this environment [23]. Finally, the ISO/IEEE 11073 Service-oriented Device Connectivity (SDC) is a set of communication standards designed to enable manufacturer-independent medical device interoperability in the OR [24]. Like MDPnP, it uses web services and is based on an SOA. A comprehensive overview and comparison of the presented projects and protocols can be found in [22].

### 2.2. Safety and Security in Life Cycle Processes

**Figure 1:**
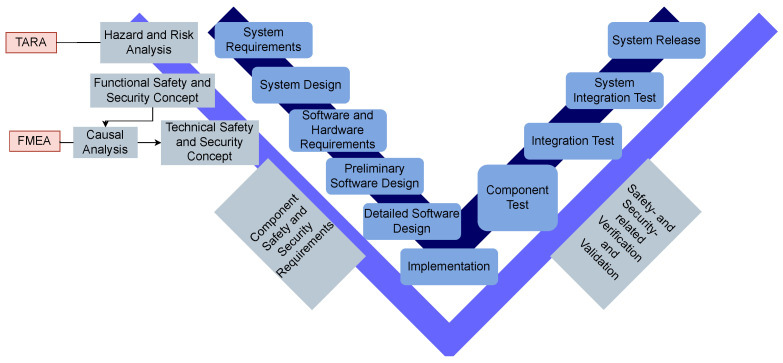
Safety and security analysis alongside V-model development.

There are several development models, but one of the most popular in software-dominated industries is the V-model [25] (Figure 1). It provides a structured approach to the development of systems, including mechanics, electronics, and software. An analysis of the requirements and specifications for the system is the first step in the application of the model. Afterwards, the development, integration, and validation of the mechanical, electronic, and software-based components of the system are executed.

Alongside the formulation and discovery process of requirements, the elicitation of a hazard and risk analysis, as well as functional and technical analyses addressing safety and security, are conducted. The goal of these analyses is to identify risks and threats in order to define requirements for system development. In the automotive industry, models for the analyses conduction have been established, such as TARA and Failure Mode and Effects Analysis (FMEA).

A comprehensive cybersecurity process is proposed by the National Institute of Standards and Technology (NIST) *"Framework for Improving Critical Infrastructure Cybersecurity"* [26], which is recommended and adapted by the U.S. Food and Drug Administration (FDA) guidance documents [10,27]. Medical device manufacturers should assess and address the risks posed by vulnerabilities in their devices, considering the magnitude of the problem and the risks encountered. They should also evaluate the residual risk, benefit/risk ratio, and risk introduced by the remediation. Changes to address controlled risk vulnerabilities are generally considered as product improvements and not recalls by the FDA. Therefore, routine cybersecurity updates are usually considered as device enhancements. Ref. [27] Five core functions (Identify, Protect, Detect, Respond, and Recover) should be adopted and utilized [26,27,28], and Draegerwerk has implemented a cybersecurity process that includes similar actions [29]:

Identify: Manufacturers should define the security and key performance characteristics of their products and the potential severity of patient harm in the event of a compromise, and use threat models to assess the exploitability of vulnerabilities and determine the effectiveness of proposed or implemented remedies. Additionally, they should also analyze various sources of quality data, actively seek out and address sources of cybersecurity signals, and develop strategies to improve their ability to detect them. The activities of the identification function are essential for the other functions and can be considered as the basis of the framework.

Protect: Supporting the ability to contain the impact of a cyberattack is the objective of this function. Manufacturers should characterize and assess identified vulnerabilities, conduct cybersecurity risk analyses and threat modeling for each of their devices, and update these analyses over time. Furthermore, they should implement countermeasures such as IAM or awareness training for users.

Detect: Manufacturers should analyze possible threat sources and consider incorporating design features that enhance the device’s ability to detect threats and produce forensic evidence in the event of an attack. They should also have a process in place to assess the impact of a cybersecurity signal on all devices within their product portfolio and on specific components within a device.

Respond: Medical device manufacturers should implement device design controls to take action in case of a detected cybersecurity incident. They should assess and provide users with compensating control mechanisms to mitigate the risk of patient harm and ensure the cybersecurity of their devices. Manufacturers should address identified cybersecurity vulnerabilities by developing and implementing remedial actions.

Recover: Manufacturers should take steps to support the timely restoration of normal operations to minimize the impact of a cybersecurity incident. This can include the timely delivery of security updates. Moreover, they should inform users and implement a coordinated vulnerability disclosure policy and practice.

In this paper, we focus on the identification of vulnerabilities in medical devices.

### 2.3. Threat Modeling

To model security analysis, different automotive-specific and non-industry-specific approaches exist. According to Figure 1, the security analysis is part of the system’s requirement step in architecture development. TARA is one method used to identify security risk and is based on an attacker-centric approach. Premised on historical information such as incident reports or contemporary security measures, threats are analyzed. After their identification, the methods and objectives of the potential attackers are listed and the exposure and vulnerability toward these risks are identified and documented. The risks are assigned with necessary protection procedures and compared to those existing in the company. Thereby, the security strategy and development steps are pointed out [30].

TARA and FMEA are two different methodologies for security-related risk analysis and risk management. While the TARA takes place in an early development phase for system requirements formulation, the FMEA focuses on identifying and evaluating potential failures and their impacts on a system thereafter (Figure 1). These requirements and the system design that resulted thereby is the basis of the FMEA. It is used with the goal of identifying and evaluating potential failures within the system or product.

The Process for Attack Simulation and Threat Analysis (PASTA) [31] is a risk-based threat modeling framework that aims to integrate business objectives and technical requirements, involve key decision makers, and produce an asset-centric output in the form of threat enumeration and scoring. PASTA consists of seven stages of analysis, including defining objectives and technical scope, decomposing the application, conducting a threat and vulnerability analysis, modeling attacks, and analyzing the risk and impact. To facilitate these stages, PASTA employs various design and elicitation tools, such as high-level architectural diagrams, Data Flow Diagrams (DFDs), attack trees, and use and abuse cases. PASTA is widely recognized as a risk-based framework that adopts an attacker-centric perspective.

The Operationally Critical Threat, Asset, and Vulnerability Evaluation (OCTAVE) [32] is a risk-based approach to cybersecurity assessment and planning that aims to evaluate organizational risks and identify vulnerabilities in an organization’s information infrastructure. It consists of three phases: building asset-based threat profiles, identifying infrastructure vulnerabilities, and developing a security strategy and plans. OCTAVE was originally designed for large organizations, but a version called OCTAVE-S has been developed specifically for small organizations. While the method is comprehensive and flexible, it requires a significant time commitment and the documentation can be large and vague [33]. There are plans to update OCTAVE, which may address these issues [34].

In this context, further methods exist. The STRIDE model (Spoofing, Tampering, Repudiation, Information disclosure, Denial of service, and Elevation of privilege) is a qualitative approach by Microsoft using a system’s DFD as the base for an evaluation [35]. Security-related system properties are labeled and checked regarding security characteristics, and threats are identified.

Automotive-specific methods that are based on the STRIDE model are HEAling Vulnerabilities to ENhance Software Security and Safety (HEAVENS) for all systems of the E/E architecture and Security Aware Hazard Analysis and Risk Assessment (SAHARA) for embedded systems. The SAHARA model checks for confidentiality, availability, and integrity attributes and enables threat and risk identification to extract a threat level and a security level [36]. HEAVENS is an approach combining Microsoft’s STRIDE with Evita [37], a further attack-scenario-based method. HEAVENS includes authenticity, authorization, non-repudiation, privacy, and freshness, on top of the previously mentioned attributes. It evaluates the whole E/E architecture and provides a risk matrix as a result that includes threat as well as impact levels, but also high-level security requirements [35]. The extension HEAVENS 2.0 is improved according to gaps that could be identified when comparing HEAVENS 1.0 to the requirements of ISO/SAE 21434. It includes an attack path analysis and risk treatment decisions with the result of identifying cybersecurity goals, and claims to be compliant with the regulation [13]. HEAVENS 1.0 and 2.0, as mentioned in [13], have the potential to be used in industries with similar characteristics, such as the medical device industry, with some slight modifications.

There are more threat-modeling approaches than those described here and each of them has its dedicated application area. Shevchenko et al. provide a comprehensive overview of twelve different threat-modeling methods [34].

### 2.4. Safety and Risk Classification for Medical Devices

**Figure 2:**
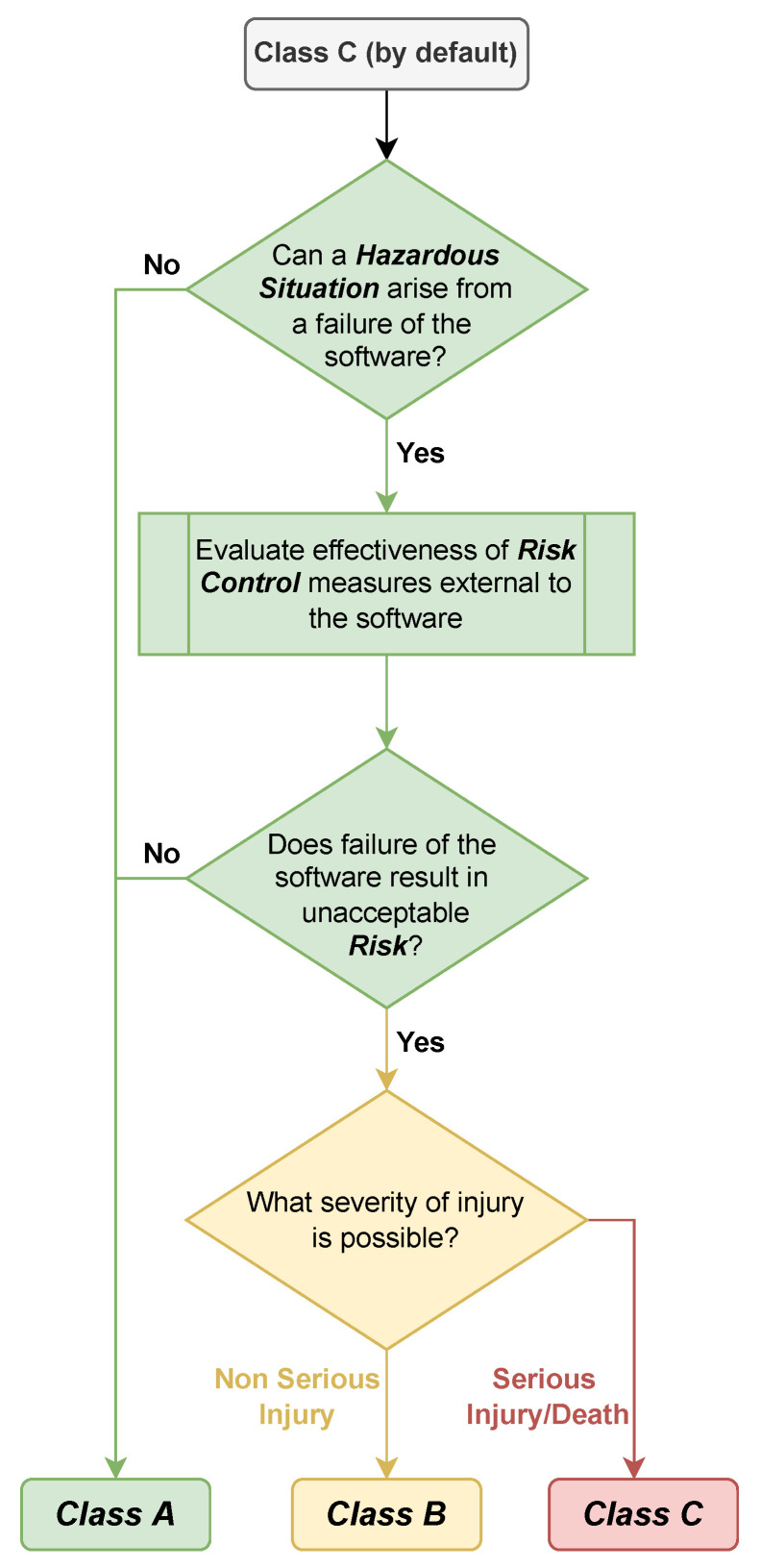
Software safety classification according to IEC62304 [38].

According to standard IEC 62304 [38], the software can be classified into three categories based on the potential risk level that it poses (Figure 2). Class A software poses the lowest risk, and can only be classified as such if no hazardous situations can occur due to software errors, or if any hazardous situations can be adequately controlled to prevent unacceptable risks. If the measures put in place to control risk are not sufficient to prevent unacceptable risks, the software is classified as B if it could potentially cause non-serious injuries, or C if it could potentially cause serious injuries or death. An injury is considered serious if it requires medical intervention to prevent permanent harm or is life-threatening. Any risk that could result in serious injury is considered unacceptable. Furthermore, the IEC 60601-1 standard for medical devices [39] requires that the devices are designed in such a way as to prevent the first failure of a system from causing significant risks. This means that the device should be designed with sufficient safeguards and redundancy to ensure that a single failure or malfunction will not result in an unacceptable level of risk to the patient or user.

The European Medical Device Regulation (MDR) 2017 [40] provides guidance that determines the class of a medical device based on its risk profile. The classification of a medical device determines the level of regulatory oversight and the requirements for conformity assessment and market surveillance. Whereas the MDR classifies into four different categories, the FDA uses three different categories (Table 1). The classification of a product is based on the type of product and the risk that it poses to patient health. It helps to determine the necessary regulatory requirements for each product and to ensure safety and effectiveness. The risk criteria are metrics such as the application time or degree of invasiveness.

The provided examples (Table 1) are meant to be general and are not intended to be exhaustive or definitive, as the classification of a medical device can vary based on its specific characteristics and intended use. The classification is therefore always determined for a specific, individual product [41]. In addition, if a medical device controls data from a higher-classified device in an interoperability case, it inherits that classification according to MDR [40].

### 2.5. Medical Device Standards and Regulations

The IEC 62304 [38] is a standard that specifies the software development process for medical device software, including requirements for the design, testing, and validation of software. According to the IEC 62304 [38] standard, medical device manufacturers are required to implement a risk management process in accordance with ISO 14971 [42]. The FDA also recommends using the qualitative severity levels outlined in ISO 14971 to assess the impact on health when evaluating the severity of risks [3] (Table 2).

The MDR and relevant standards such as ISO 13485 [43], ISO 14971 [42], and ISO 24971 [44] outline specific requirements for risk management in the life cycle of medical devices. For safety risks, FMEA is a tool that has been commonly used by medical device manufacturers for risk management [45], but it does not meet all of the requirements on its own and is not designed for security risk analysis. While the term “risk” is defined differently in ISO 14971 and in FMEA, it can still be useful for risk management when used in combination with other tools and methods [46]. In the course of this paper, only the Software Failure Mode and Effects Analysis (SFMEA) will be of relevance.

After the identification of risks, a risk matrix (Table 3) can be used to determine a software item’s safety classification (Section 3.1) by mapping its risk and function to a severity level, which is then used to assign the classification. In this case, this can be, as an example, low risk corresponding to class A, medium risk corresponding to class B, and high risk corresponding to class C.

IEC 81001-1 [47] and IEC 81001-5-1 [16] provide guidelines for the management of cybersecurity in healthcare technology. IEC 81001-1 is the general introduction to the IEC 81001 series and provides an overview of the principles and concepts related to cybersecurity in healthcare technology. It outlines the scope and purpose of the series of standards, as well as the main terms and definitions used in the standards. IEC 81001-5-1, on the other hand, provides specific guidance on how to manage cybersecurity risks in healthcare technology. It outlines a structured approach for identifying and evaluating cybersecurity risks, implementing protection measures, and responding to and recovering from cybersecurity events. It also provides recommendations for the design and development of secure healthcare technology, as well as for the procurement, maintenance, and decommissioning of such technology.

The IEC 81001-5-1 complements the IEC 62304 [38] with cybersecurity requirements. Together, these standards provide a comprehensive framework for ensuring the safety and effectiveness of medical devices by addressing both cybersecurity risks and the software development process. They can be used in conjunction with each other to ensure that medical devices are developed and used in a way that is safe and reliable for patients and users.

The IEC 81001-5-1 standard does not explicitly require the use of a TARA, but it is recommended by IEC 81001-5-1 to follow the processes outlined in the IEC 62304 standard, which addresses safety in the life cycle process, in order to identify the necessary activities for implementing security measures. Thus, by applying common TARA processes, it is possible to address both standards simultaneously.

**Figure 3:**
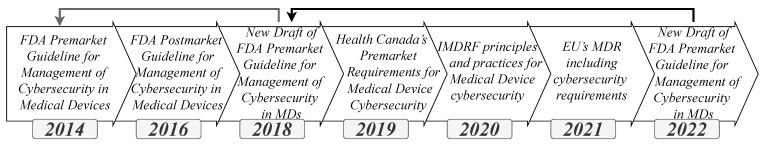
Updated evolution of medical device cybersecurity regulations based on [51,52].

Due to the rapidly changing environment in the medical device industry, the FDA released premarket guidelines in 2014, a draft update in 2018, and another draft update in 2022 (Figure 3). The new revision recognizes the need for a continuous, iterative approach to device cybersecurity throughout the product life cycle [10]. One of the articulated security objectives in these guidelines pertains to the ability to secure and timely update and patch devices. The FDA’s proposed security risk management strategy for the product life cycle also advises manufacturers to have the necessary resources to identify, assess, and mitigate cybersecurity vulnerabilities as they emerge throughout the device’s lifespan. Documentation that is updated throughout the product life cycle, such as threat models, can facilitate the rapid identification of the impact of vulnerabilities once a device has been released, and can support timely Corrective and Preventive Action (CAPA) activities [10]. To support manufacturers in the creation of threat models, the FDA funded the “Playbook for Threat Modeling in Medical Devices” [48].

The FDA postmarket guideline [27] recommends that manufacturers proactively address cybersecurity risks in their products and monitor, identify, and address any vulnerabilities or exploits as part of their postmarket management. The guidelines also outline a risk-based framework for determining when changes to address cybersecurity vulnerabilities in medical devices should be reported to the FDA and specify circumstances in which the agency does not require advance notification or reporting. The guidelines recommend that manufacturers assess the risk of patient harm based on the likelihood of exploitation, the impact of exploitation on the device’s safety and essential performance, and the severity of patient harm if exploited.

The International Medical Device Regulators Forum (IMDRF) has released a draft document outlining principles and practices for medical device cybersecurity [49]. The aim of this guidance is to identify and mitigate potential risks to patient safety by analyzing the impact of cybersecurity threats on the device performance, clinical operations, and diagnostic or therapeutic errors, and does not address issues related to data privacy breaches or the manufacturer’s enterprise. The guideline also advises the use of a threat model for medical devices as part of risk management. As it is provided by the IMDRF, it aims to support regulatory processes by phrasing out related requirements. In contrast, the Canadian “Pre-market Requirements for Medical Device Cybersecurity” [50] only mandates a risk analysis and management for certain high-risk medical devices. Furthermore, the MDR calls for cybersecurity risk management according to the state of the art, which is not further elaborated, without mentioning threat modeling [40].

In addition, privacy in terms of HIPAA becomes a greater concern for medical device manufacturers (Section 1), as the HIPAA has the purpose of establishing national standards for the protection of PHI. HIPAA applies to all entities that handle PHI, including healthcare providers, insurance plans, and healthcare clearinghouses. HIPAA requires these entities to implement appropriate physical, technical, and administrative safeguards to maintain the confidentiality, integrity, and availability of PHI. Additionally, it includes provisions that allow individuals to access, modify, and control the use and disclosure of their PHI [2].

### 2.6. Automotive Systems Evaluation and Safety Integrity Level

In the automotive industry, the development of applications requires the functional safety evaluation thereof. The standard ISO 26262 “Road vehicles—Functional safety” defines the product development processes that must be followed depending on the criticality of an application [53]. ISO 26262 classifies four different Automotive Safety Integrity Levels (ASILs) based on the risk exposure, severity, and controllability (see Table 4). The levels are Automotive Safety Integrity Level (ASIL) A, ASIL B, ASIL C, and ASIL D, where D defines the highest level of initial hazard and A resembles the lowest [54]. The additional Quality Management (QM) level represents a category where systems or components can be managed by established QM methods. The ASIL level is accompanied by goals for the identified hazards according to system safety requirements. It is necessary to prove the fulfilment of safety requirement compliance during architecture development, which is especially important due to the multilayered supplier structure typical in the industry [55].

### 2.7. Automotive Standards and Regulations

For safety analysis and quality management, the FMEA is often used throughout several industries. In the automotive industry, the FMEA is part of the standard SAE J1739 as *Potential FMEA including Design FMEA, Supplemental FMEA-MSR, and Process FMEA*. As shown in Figure 1, the FMEA is part of the system’s design in architecture development and is based on functional safety. The method is used to evaluate the potential of a failure of a process, a system, and subsystems, services, or designs [56]. The goal is to identify risks and problems that result in the deviation of a specific function from its intended functionality. In order to achieve that, the FMEA is used to identify the types of failures and their causes and effects to determine, evaluate, and reduce risks. The steps involved are structural analysis, functional analysis, failure analysis, risk analysis, optimization, and documentation [57].

Updating automotive application software, firmware, or other software packages within the Electric/Electronic (E/E) are prevailing challenges to be solved and secured in the industry [58]. While some OEMs have already demonstrated SOTA updates [59], there is no standardized procedure thus far. Challenges to be solved are related to safety, security, and privacy. In addition, there is uncertainty regarding process frameworks to comply with standards relevant to the release of an update [60].

Related to SOTA updates and automotive cybersecurity, the drafts of the UN vehicle regulation 155 and 156 [61] were published. In Regulation 156, SUMSs are defined as process models for update delivery. These systematic approaches applied by OEMs need to be certified to fulfill security requirements in the SOTA update context. Furthermore, a risk assessment as well as methods related to cybersecurity attacks are specified in Regulation 155. It defines a CSMS as a legislative prerequisite for every vehicle OEM. It requires the OEMs to ensure and document all demanded processes and the capabilities in the near future to be certified by authorities. These regulations are not yet mandatory.

The prevailing trends are followed by an architecture evolution toward dynamic SOAs or mixed architectures that combine signal-based and service-oriented communication. A key challenge to be solved is to realize SOTA updates to regularly add functions but also to update security countermeasures. For the latter, security-related measures from IAM over intrusion detection and firewalls are required [62]. Focusing on automotive updates, UN Regulation No. 156 [61] specifies certificates and general documents for update conformity within the industry. This standard, as well as the ISO 24089 [63] draft, describes requirements for update engineering and approval. The OTA update development is specified and several recommendations are given. Still, there is no detailed update development and deployment process model to serve as a blueprint for integrating update engineering for OEMs.

In the automotive industry, safety and security have become more and more important, not only over the vehicle life cycle but already during development. Approaches to prevent threats in the context of functional safety and cybersecurity are gaining importance. Since 2011, the ISO 26262 standard has provided guidelines for automotive safety, while the automotive security guideline SAE J3061 was published in 2016 [64]. The latter recommends the usage of TARA methods. These models are supposed to discover threats, assess the risk of these threats, and analyze a risk level accordingly.

Besides the SAE J3061 Cybersecurity guidebook, the ISO/SAE 21434 regulation defines an automotive-specific cybersecurity engineering standard concerning the whole vehicle life cycle [65]. A key aspect of the standard is the TARA, which is used to identify security risks and threats, with the purpose of developing countermeasures and mitigation strategies [13].

## 3. Related Work

### 3.1. Medical

To address vulnerabilities in medical devices that monitor patients’ vital signs, Luckett et al. suggested using attack graph modeling to identify these, assess risks, and develop strategies for protecting medical devices from attackers [66]. The researchers examined common vulnerabilities and attack strategies related to these devices, including Bluetooth-enabled sensors and Android applications. They provided an example of attack graph modeling for a theoretical device to highlight vulnerabilities and potential mitigation techniques for designing similar devices.

Since the integration of SOA in the automotive and medical industries is increasing, the shift in communication patterns will also impact information security measures. In [67], the authors compared different SOA protocols in these industries and explained the underlying communication patterns, showing that both domains can exploit synergies. They also presented a methodology for developing an SOA-based Intrusion Detection System (IDS) by deriving relevant features. Furthermore, they contributed to the understanding of SOA protocols and their potential use in proposing an IDS for both the automotive and medical industries. Based on a use case for medical devices in an OR connected via SDC, the authors analyzed threats in network communications in the context of anomalies.

Vakhter et al. provided an elaborate overview of threat modeling applicable to miniaturized wireless biomedical devices and proposed a domain-specific qualitative and quantitative threat model [68]. This threat model focuses on noninvasive direct attacks against telemetry interfaces and uses them for risk analysis.

In their position paper, Sion et al. discussed the strengths and weaknesses of security threat modeling that is based on DFDs, and motivated their research with a DFD for an Health Information System (HIS) [69]. Despite advantages such as technology independence, complexity management, and simplicity of notation, they pointed out disadvantages such as a single level of abstraction, data modeling only by labels, and a lack of a set of security concepts.

Ahmed et al. provided an evaluation model for the cybersecurity of hospitals [4]. The goal of their research was to create a model that helps healthcare facilities understand and assess their current cybersecurity status, identify potential risks, and implement measures to mitigate those risks. This model can be used as a tool to help hospitals understand their current cybersecurity situation and make informed decisions about how to improve it. In addition, proposed cybersecurity measures can be incorporated into the design of new healthcare facilities before they become operational.

In [70], the authors proposed a use case approach for assessing the cybersecurity and privacy requirements of Point of Care (POC) medical devices. Furthermore, they detailed the use case approach in the context of a real healthcare IT infrastructure that includes various components, such as an HIS, application servers, and medical devices, as well as interactions with different participants. This approach can also be used to analyze cybersecurity and privacy risks in various threat scenarios and provide information for decision making and regulatory compliance. POC medical devices are typically used by clinicians to provide near-patient care and/or diagnosis and treat many patients after appropriate preparation. In contrast, Personal Health Devices (PHDs) are used in a private or domestic setting by a single person and are generally assigned to that person (Ref. [22]). Nevertheless, Jofre et al. focused on smartphone apps as POC devices, which should be rather considered as PHDs in the sense of this article and the clinical Information Technology (IT) infrastructure.

In this research, we focused on the analysis of POC medical devices in OR, an area that has not been adequately addressed in previous studies. Many of these previous studies have concentrated on wearable and PHDs or the clinical IT infrastructure, but have not included a formal process using a TARA approach or considered necessary medical standards for safety and security. The authors aim to fill this gap in the literature by examining POC medical devices in OR networks and considering these important factors. The results can also be applied to other areas of the hospital, such as Intensive Care Units (ICUs).

Fernandes et al. investigated the use of techniques based on Threat Artificial Intelligence, Chaos, Entropy and Security (TAICE&S) for solving cybersecurity problems in cryopreservation laboratories [71]. Their research aimed to address General Data Protection Regulation (GDPR) issues in this type of laboratory using techniques derived from the relationship between TAICE and cybersecurity. In addition, the authors used logic programming and AI-based techniques for knowledge representation and reasoning, as well as artificial-neural-network-based computational frameworks. They also included a case study of data collection and processing on security policies in cryopreservation laboratories.

Radanliev et al. proposed a concept for a healthcare system supported by autonomous artificial intelligence (AutoAI) [72]. The aim was to use edge health devices with real-time data to prepare and adapt the health system for future pandemics. The authors developed two scenarios for the application of cybersecurity with AutoAI, namely a self-optimizing predictive cyber risk analysis of health system failures during a disease X event, and a self-adaptive prediction of medical production and supply chain bottlenecks during future pandemics. These scenarios were developed to address the logistical challenges and disruptions of complex vaccine distribution production and supply chains with optimization algorithms. The new methodology presented in this paper provides a practical application for designing a self-optimizing AutoAI capable of predicting cyber risks in healthcare systems through real-time algorithmic analysis. Furthermore, it can be applied to the design of a self-adaptive AutoAI specifically suited for predicting bottlenecks through the autonomous analysis of digital healthcare systems. The authors highlight the need for interdisciplinary research to address concerns about IoT risks and security and propose solutions that promote the safe development of digital health systems by integrating AI algorithms into vaccine supply chains and cyber risk models.

Silvestri et al. conducted a study using machine learning models to analyze natural language documents related to healthcare cyber threats and vulnerabilities [73]. Using BERT and XGBoost neural language models for a threat and vulnerability analysis, the authors conducted experiments using cybersecurity news from Hacker News and Common Vulnerabilities and Exposures (CVE) vulnerability reports. In addition, they demonstrated the effectiveness of the proposed approach, which provides a realistic way to assess threats and vulnerabilities using natural language text, and enables it to be applied in real-world healthcare ecosystems. It also recognized the challenges of analyzing threats and vulnerabilities in healthcare due to a large amount of unstructured natural language data and the complexity of the language used in cybersecurity.

### 3.2. Automotive

In the automotive industry, security risks related to highly connected vehicles and V2X have received much attention for several years [74,75]. The threats include the risks caused by attacks on the vehicle network [76]. An external party gaining access to this network may also cause deaths or massive damage [77]. The focus of prevalent research is therefore on generating security by design in E/E architecture development to minimize the risk of attacks. Standards such as the ISO 26262 [53] and the influence thereof on SOTA are studied in [78,79,80] among others. Security risks permanently increase in the context of Advanced Driver Assistance Systems (ADAS) or V2X, bringing it to the fore of research [76]. With the purpose of extracting potential risks and vulnerabilities for vehicles and E/Es, security analysis models exist, e.g., [81]. One of the numerous models and frameworks for automotive TARA is known as the HEAVENS security model [81] (Section 2.3).

In 2017, an analysis was conducted by Kreissl [82] to assess the security of the Scalable service-Oriented MiddlewarE over IP (SOME/IP) protocol within an automotive onboard communication system. This evaluation identified a total of 18 potential threats within an automotive onboard communication system using SOME/IP. Using the HEAVENS [83] risk analysis method, 11 of these threats were classified as high risk and 3 as a critical risk. The main issue identified was the lack of security features in SOME/IP, leading the author to propose various use cases and associated security properties, as well as discuss potential security mechanisms to address these issues.

## 4. Threat and Risk Assessment (TARA) Adoption

**Figure 4:**
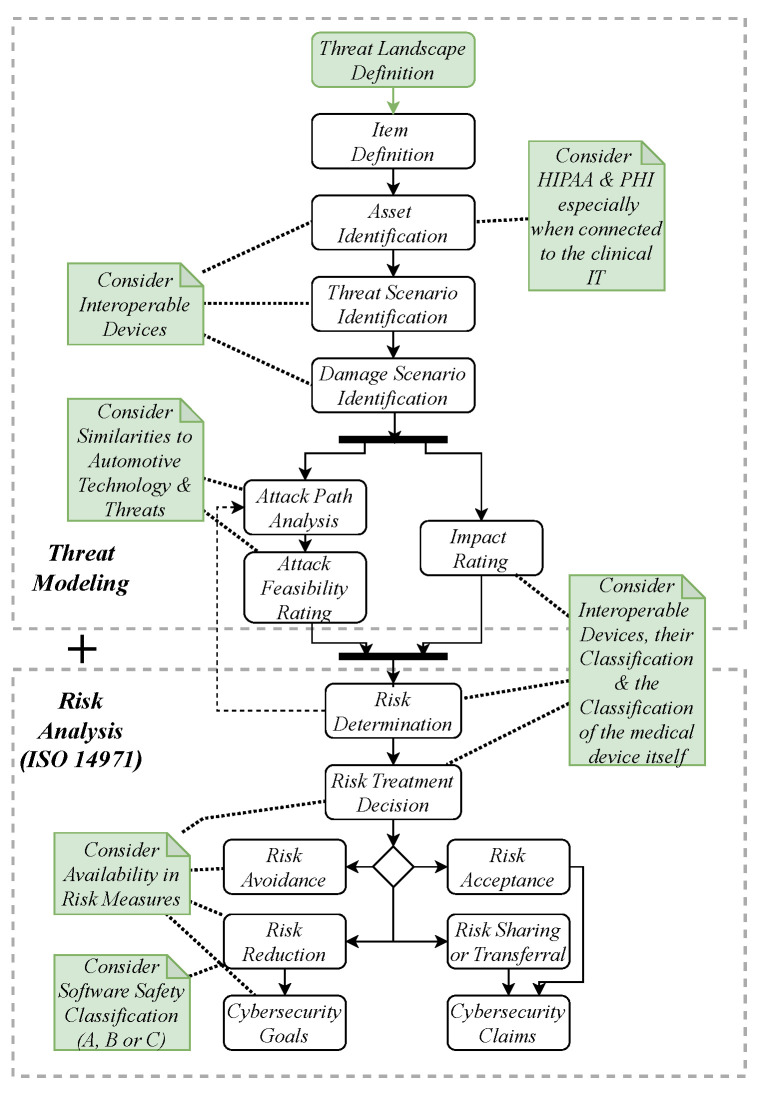
Heavens 2.0 workflow [13] considering a medical context (Section 4.1).

HEAVENS 1.0 has been successfully implemented in the automotive industry, which has comparable safety and security requirements to the medical device industry. Since HEAVENS 1.0 has certain shortcomings, such as counter-intuitive threat values [13], a low possibility for customization, and a low process efficiency, HEAVENS 2.0 was created to address these issues. In addition, the creators of HEAVENS 2.0 declared the model as suitable for medical devices (Section 2.3). Furthermore, it fulfils the threat modeling as well as the risk analysis in a single process (Figure 4). Therefore, we chose HEAVENS 2.0 as the most suitable TARA for medical devices and chose to apply it to our interoperable medical device use case (Section 5). Both HEAVENS models are based on the evaluation of functional use cases. Based on that, the framework is used to perform a threat and risk analysis in a joint process. The output of the model execution is a risk matrix accompanied by security requirements and methods [35].

The HEAVENS application (see Figure 4) starts by defining the item under examination. The specific use case needs to be defined precisely and system boundaries are required to be set. The following asset and threat scenario identification follows the STRIDE model and uses the data flow of the specific item to identify potential threats. For HEAVENS 2.0, new steps are to be carried out after these activities. At first, attack paths for the identified threats are examined by creating attack trees to identify the root of a threat. Afterwards, a feasibility rating is associated with the threats to display the attack potential. The result of this task is an attack feasibility rating that takes into account access means, asset exposure, and the knowledge of an item, for example. At the same time, an impact rating is calculated based on safety and privacy, as well as operational and financial characteristics.

The ratings are used to conduct a risk analysis starting with a risk determination of the risk. This, in turn, is the basis for the risk treatment decision being either its avoidance, if possible, or its acceptance, reduction, sharing, or transferal. While the reduction is followed by cybersecurity goals to be determined, the reduction, sharing, or transferal results in cybersecurity claims. The latter describes statements constituting reasons for risk acceptability [13].

In conclusion, the HEAVENS 2.0 model is a comprehensive qualitative and quantitative approach to identifying risks and threats with the purpose of identifying, preventing, or reducing them.

### 4.1. Differences and Similarities in the Automotive and Medical Fields

Although the medical and automotive industries have similar safety and security risks, there are differences that make an unchanged transfer of methods not fully appropriate. A key factor distinguishing the industries is the operating environment. Whereas medical devices operate in a more static and easier-to-isolate area, vehicles are exposed to other surrounding conditions as they are moving in a rather unrestricted, open environment and interact with each other as well as infrastructure and further systems. In an OR, it is rather unlikely that unknown devices, which are not operating in the hospital network, interact with existing ones. To introduce and test new equipment in the OR, there is a commissioning process; after this, it can be used in surgery [24]. In that sense, an OR can rather be compared to a restricted area such as a car workshop for vehicles. Considering the case of SOTA updates, the necessity of in-use updates is not strictly relevant for medical devices. The controllability of the less mobile devices exposes the SOTA update process, according to the previously mentioned boundary conditions, to fewer safety and security risks than vehicles.

Real-time systems can additionally be seen as a slight difference. An example is ADAS functions. To enable highly automated driving, the vehicle systems need to fulfill strict time constraints and respond to changing environmental circumstances in real time. Regarding medical devices, Real-Time Operating System (RTOS) are necessary and in use; still, the time constraints are less strict as the connections to other devices are foreseeable.

Other distinctive features are computing units and the backend infrastructure. In a hospital, these entities can be hosted within the private network that the medical device operates in. In the automotive industry, vehicle fleets need to be controlled and protected; therefore the connection between these vehicles and a backend infrastructure happens in various public as well as private networks.

Even if there are differences between the industries, security risks that arose and cyberattacks that happened in the past are similar to a high degree. It has been observed that the technology used in the automotive and medical industries are partially similar [19]. Due to this technological overlap, it is likely that vulnerabilities that have been identified in the automotive industry could also be present in the medical field. This highlights the need for both industries to be proactive in securing their systems and protecting against potential cyber threats. This technological overlap with similar requirements, especially regarding safety and security, makes similar processes and measures applicable in both industries.

The security guidelines and standards are not as concrete in the medical field because ISO/SAE21434 has a defined TARA workflow that must be met step by step, whereas the medical device guidelines and standards only require a threat model and a corresponding risk analysis according to ISO 14971. This leaves manufacturers greater room for (mis)interpretation. It may result in potentially inadequate analyses and measures. Nevertheless, procedures and methods are proposed that are unfortunately not adapted to the medical field and mostly originate from the IT sector.

The software safety classification imposed by IEC 62304 and the corresponding process requirements are comparable to ISO 26262, although three different classes are to be distinguished rather than four different classes. However, a risk classification for the entire product, as required by the FDA and MDR, is not applied in the automotive industry. In terms of processes and methods associated with safety risks, such as FMEA, both areas appear to be at a similarly high level, with slight differences in the individual areas.

### 4.2. Heavens 2.0 in Medical Context

Due to the previously mentioned differences, some adoptions find it necessary to use HEAVENS 2.0 in the medical context. Medical devices are more heterogeneous and face different threats depending on their intended use and application. Therefore, the external threat landscape must be determined prior to the first steps “Item Definition” and “Asset Identification”. Furthermore, for an effective “Risk determination” and “Damage Scenario Identification”, the medical device class of the device itself and the connected devices needs to be taken into account. For example, the dosing of an infusion pump that is affected by a cybersecurity threat may pose a different risk in a medical devices operation than as a vulnerability in a thermometer [27].

Once the threat landscape is identified, it can be reused for other medical devices in the same context, such as devices in an OR such as OR tables or angiography systems, which are C-shaped devices for interoperative imaging with X-ray technology. The steps from HEAVENS 2.0 can generally be applied for medical devices, but the differences in automotive and medical contexts need to be considered (Figure 4).

### 4.3. Threat Landscape in Operating Rooms

The FDA recommends that manufacturers fully consider cybersecurity risks when designing devices by evaluating the potential safety and security risks within the context of the system in which the device will be used. This involves making assumptions about the system and environment, such as hypothesizing that a hospital network may be hostile and that an adversary may have the ability to alter, drop, or replay packets [10].

In the past, there have been several instances of cyberattacks and reported vulnerabilities in hospital equipment and medical devices, highlighting the importance of identifying potential threats.

Threat 1: A ransomware attack in 2016 on the Hollywood Presbyterian Medical Center in Los Angeles led to the shutdown of the hospital’s computer systems [84]. Later that year, two additional hospitals in California [85] and one in Canada [86] were targeted by ransomware attacks, and the tendency of this kind of attacks is rising (Section 1). The 2017 Wannacry ransomware attack affected specific gantry and robot imagers, as it could be transmitted through various means, such as the use of infected memory sticks or the opening of malicious emails on the system by clinicians [87].

Threat 2: In 2017, it was discovered that some cleaning and disinfection equipment could potentially be accessed and have its data manipulated during an attack on a hospital, laboratory, or practice’s internal network. A hacker could potentially exploit this vulnerability by attempting to misuse the data to gain illegal access and manipulate program control. They could also try to forge batch protocols through data analysis and knowledge of instrument preparation in order to hide any manipulations. This potential vulnerability also applies to unauthorized actions by individuals with legitimate access to the relevant network [88].

Threat 3 In 2019, insulin pumps were recalled due to the potential for attackers to remotely adjust the dosage of insulin delivered to a patient [89,90].

Threat 4: In 2019, the FDA issued a warning about a potential cyberattack on certain models of implantable cardiac devices, clinic programmers, and home monitors resulting from a wireless telemetry protocol [91].

Threat 5: In 2019, the German Federal Institute for Drugs and Medical Devices (BfArM) issued a warning about certain sterilizers, stating that an attacker could potentially manipulate the system to influence the efficiency of the sterilization process via remote access [92].

Threat 6: In 2020, the FDA issued a warning about vulnerabilities in certain models of central stations and telemetry servers, which are used to track vital signs of patients [93]. Attackers could remotely control the device and interfere with alarms, e.g., by silencing them or generating false alarms.

**Figure 5:**
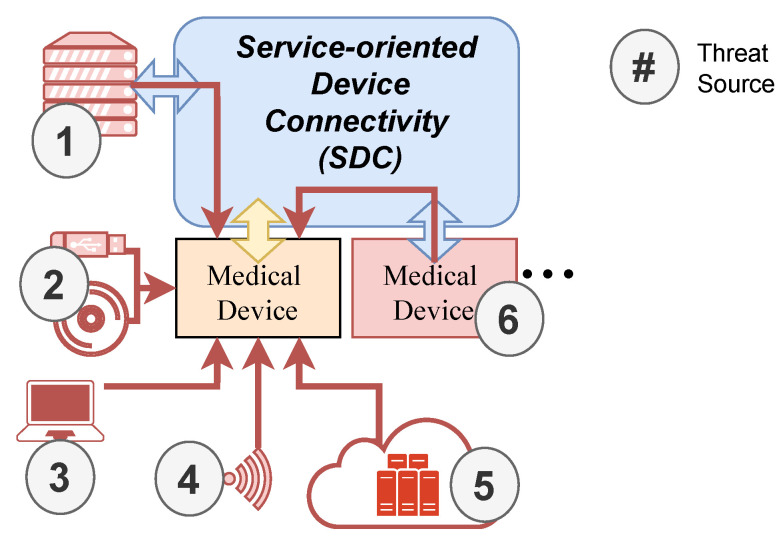
Threat landscape in an Operating Room (OR) network of medical devices based on the overall concept for SDC [94] with different threat sources (1–6).

There are other potential threat landscapes to consider, such as the manufacturing line. However, for the purposes of this research, we focused on the OR (Figure 5). By including the threats (Threat 1–6) listed before, the following threat sources can be derived:

Threat Source 1—Clinical IT-Infrastructure: In case the clinical IT infrastructure was compromised, these attacks can also affect the ORs (Threat 1, Threat 2, Threat 5).

Threat Source 2—External Storage Devices: External storage devices may introduce malware or other malicious software into the system (Threat 1).

Threat Source 3—Diagnostic and Maintenance Tools: The diagnostic and maintenance interfaces could be compromised and provide access to update and configuration functionality. In the automotive context, interfaces such as On-Board Diagnostics (OBD) represent a gateway for attackers to gain access to vehicle systems and data. In the case of SOTA updates, this can be realized by malicious malware sent to the vehicle [95].

Threat Source 4—Over The Air (OTA)-Communication: Since physical access is no longer required for OTA communication such as Bluetooth, the attack surface increases and unauthorized access from outside the OR or hospital is possible (Threat 3, Threat 4).

Threat Source 5—Backend Systems and Internet Connection: Devices in the OR may be connected to backend systems via the Internet, which could potentially provide an entry point for attackers (Threat 5).

Threat Source 6—Connection to Compromised Devices: Other connected medical devices may already be compromised. Due to a shared attack surface, this compromise could spread to other devices (Threat 1, Threat 5, Threat 6).

Manufacturers of devices that have not previously processed patient data may now also need to consider compliance with the HIPAA in case they are theoretically able to process the data in a SDC network. In addition, devices in this network will need to be compliant with HIPAA in order to establish a connection through SDC to the clinical IT infrastructure.

## 5. Heavens 2.0 Use Case

With the purpose of demonstrating the security model HEAVENS 2.0 in the medical context, we use the requirements and use case as described in [19]. It can be described as an interoperable, flexible OR table for the run-time adaption of other medical devices. It is based on a mixed E/E architecture incorporating service-oriented and signal-based communication. For external communication, an SDC interface is considered with which network participants may control the OR table motion, as well as read its current joint positions. The subsequent threat landscape is described in Section 4.

### 5.1. Data Flow Diagram for Item Definition and Asset Identification

**Figure 6:**
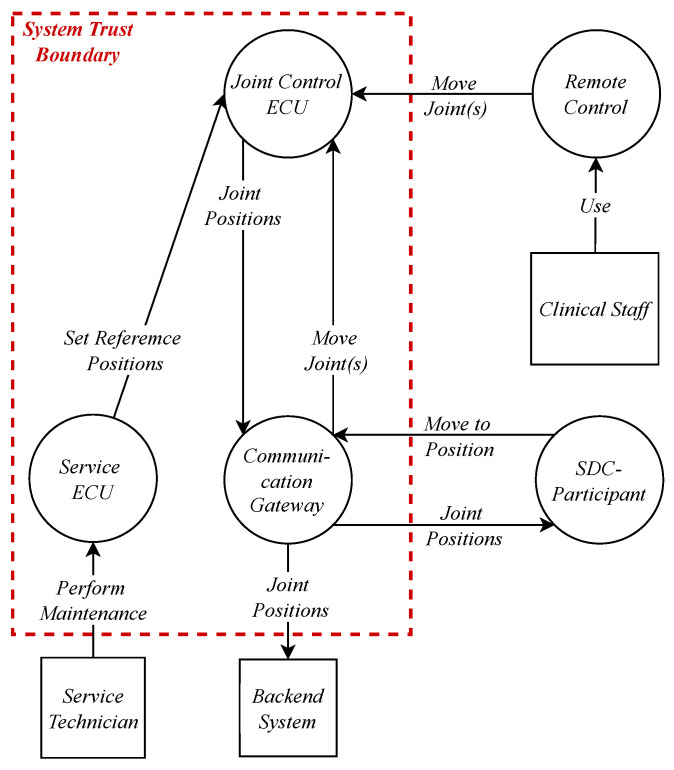
DFD for joint positions of an interoperable OR table.

Based on the architecture presented in [19], we performed a per-element STRIDE with a DFD (Figure 6). ECUs controlling joints of the OR table are handled generically here since their main task is the control of their joint positions. Furthermore, the reference positions can be set by a service technician via a service ECU. A movement for the joint can then be either invoked via remote control by the clinical staff or by an SDC participant such as an angiography system. The resulting positions are then communicated to consumers in the SDC network.

### 5.2. Threat and Damage Scenario Identification

**Figure 7:**
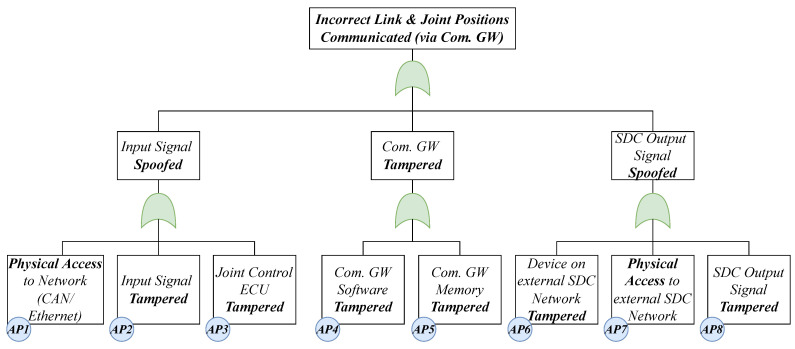
Attack tree for communication of incorrect link and joint positions.

Since a high-risk results from the communication of erroneous positions causing collisions or asynchronous movements with other devices, we chose it as a damage scenario. There are various possible attack paths that could be used in this scenario (Figure 7). An attack path is a series of steps or actions that an attacker takes to exploit vulnerabilities in a system or network to gain unauthorized access. For example, an incorrect position could be communicated if the Communication Gateway (Com. GW) signal input is spoofed once the attacker has physical access to the system’s internal network (AP1).

**Figure 8:**
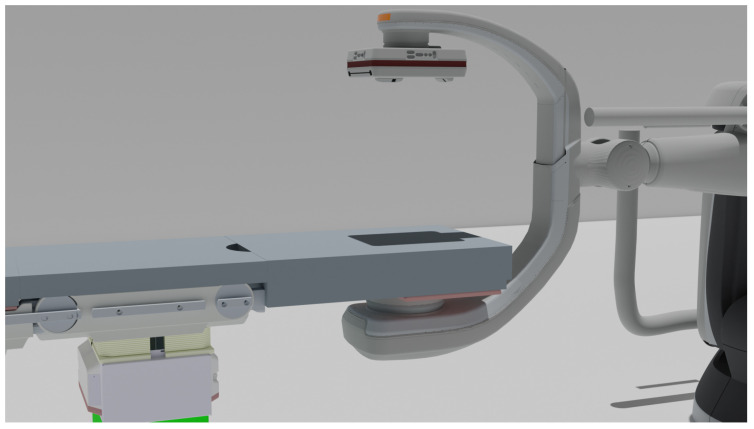
OR table collision with an angiography system.

If the height of the OR table is incorrectly communicated to a connected device such as an angiography system, serious damage can occur to both systems or even the patient in the case of a collision (Figure 8).

### 5.3. Risk Assessment

Based on the sub-parameters *expertise, knowledge of item, window of opportunity, and equipment* proposed by Lautenbach et al. [13], the Attack Feasibility Rating (AFR) can be determined (Table 5). In attack path AP1, for example, only some specialized equipment, as well as proficient expertise, is required to open the system and connect to the physical network. However, the window of opportunity is very small since the attacker needs to obtain access to the hospital and ORs, which is very restricted to the clinical staff. Furthermore, the knowledge of the item is not publicly known as manufacturers protect their development documents and the corresponding source code. To calculate the final AFR, the normalized sum for all sub-parameter values *a* can be calculated as follows [13]:

(1) $$A_{sum}=\frac{w_{x}a_{x}+w_{k}a_{k}+w_{w}a_{w}+w_{e}a_{e}}{3\cdot (w_{x}+w_{k}+w_{w}+w_{e})}$$

This example with equal weighting ($w_{x}=w_{k}=w_{w}=w_{e}=1$) and a range from 0 to 3 for each parameter *a* leads to an AFR of 42% for attack path AP1.

### 5.4. Treatment Decision

Table 6 sums up the risk values for the different threat scenarios based on the AFR and impact rating of the threat scenarios. The AFR of a threat scenario is based on the corresponding attack path with the highest AFR, and the impact rating is severe in all three cases as an erroneous position information of the OR table may lead to the serious harm of the patient (Section 5.2). Thus, each of the examined threat scenarios has a risk value of 5.

Software measures for security threats should be assigned an appropriate software safety classification based on their risk level (Section 2.4). For instance, risk values from 1–2 can be treated as class A, 3–4 treated as class B, and 5 treated as class C.

### 5.5. Cybersecurity Goals

The cybersecurity goals result in the following requirements (see also [19,67]):

**R1**  *Security properties (authenticity, integrity, confidentiality) must be ensured for network communication.*
**R2**  *Patient harm resulting from the misuse of connectivity interfaces needs to always be avoided by the system.*
**R3**  *The system must detect if the communicated joint positions are plausible.*
**R4**  *The system must be able to detect unknown attacks.*

The inclusion of the expected connected devices and the purpose of the data that they consume are critical in determining risk. Clinical decisions or the behavior of other systems, such as the movement of an angiography system, may depend on the published data. Thus, it should always be determined in what context a medical device, and, in particular, its external interface, has been evaluated. This is partly in contrast to the Plug and Play (PnP) vision of projects such as SDC or MDPnP. Due to the long life cycles of medical devices in OR, it is imperative for future networked medical devices to adapt the design and documentation accordingly in order to PnP.

### 5.6. Relation to SFMEA

The DFD (Figure 6) was used here as an input for a SFMEA. Table 7 lists exemplary risks resulting from failures in the system. Based on Table 3, a risk determination can be applied to the risks identified in Table 7. For example, R3 may happen due to data corruption in the service or signal of the joint positions provided by the joint control ECUs. A simple risk mitigation for this kind of failure is a second channel, such as a Cyclic Redundancy Check (CRC), that is sent along the payload. Failures during the parsing of incoming services/signals (P1) nevertheless cannot be detected by these measures, since the same value is sent over and over again during a movement. Thus, the positions must be checked for plausibility in the system context, e.g., a static position is not possible if the motors are moving.

Safety measures can also serve as security measures and vice versa. Thus, the previous example for position plausibility can be used for checking abnormal behavior during an ongoing attack resulting in implausible joint positions. Anomaly detection, such as through the use of an anomaly-based IDS, can also be used to identify these kinds of anomalies caused by system failures, as demonstrated by Grimm et al. [96]. In the use case examined here, by supervising the plausibility of the joint positions, security and safety risks can be mitigated. This can be achieved, e.g., by using redundant sensors and/or sensor fusion, or by checking the plausibility of signals with static or machine learning checks as proposed by Weber et al. [97].

## 6. Results and Future Work

We have shown the suitability of the HEAVENS 2.0 TARA from the automotive domain for interoperable medical devices in the OR based on a threat landscape derived from known vulnerabilities and attacks in the medical field. In addition, this approach helps to meet the key requirement of threat modeling with appropriate risk analysis and management, which is required by the vast majority of medical device standards and regulations. This contributes mainly to the first step (identify) of the NIST cybersecurity framework. Since the attack path analysis is rated as quite time-consuming by [13], the corresponding TARA needs to be extensive before the product release in order to be able to react more quickly to security events.

It should be noted that the risk analysis provided does not purport to be comprehensive as per ISO 14971 standards, as it primarily focuses on identifying and analyzing the risks associated with security-related software and how they relate to the safety-related risks present in the software, as well as the corresponding software safety classification. It does not cover risks arising, for example, from the unintended or improper use of the equipment as required by the standard.

To follow the NIST cybersecurity framework, the next steps are the protection from and detection of cyberattacks (Section 2.2). However, complicated processes such as safety or security measures may result in even more harm to the patient due to decreases in availability and greater stress toward the clinical staff. Efforts to improve security in health IT systems following a breach can introduce changes in clinical work environments, potentially disrupting patient care processes and leading to a decreased quality of treatment [98]. Thus, protection cannot be achieved simply by applying known security measures from the IT sector, such as password authentication. Furthermore, as security threats evolve over time (Section 1) and medical equipment in the OR often have a life cycle of over 15 years [19], preparation is needed for new and unknown threats. A common approach for detecting unknown attacks is anomaly-based IDS [99], which is also a proposed measure by the FDA [10]. Therefore, both protection and detection measures must be properly coordinated. Furthermore, the detection of anomalies in particular can also contribute to safety in the event of system malfunction.

The use of legacy devices in hospital networks can pose security risks due to their long life cycle. For modular devices such as OR tables, this also involves legacy modules used over several product generations [19]. According to the IEC 62304 standard [38], it is important for medical device manufacturers to implement risk management measures when using legacy software. This includes incorporating the software into the device’s overall architecture and evaluating and addressing any potential security hazards through appropriate risk control measures.

In the automotive industry, a recent challenge connected to safety and security risks is SOTA updates and the development and testing thereof. Process models, data-sharing methods, and security measures need to be established adequately to ensure a safe and secure realization of these updates [62,95]. In the medical industry, it still needs to be evaluated how important the wireless updatability of devices will become. As the environment is much more delimited and device access is closer location and network-wise, the continuity of local and wired updates may be safer and more secure in the near future.

## Figures and Tables

**Table 1:** Medical device classification according to FDA and MDR.

Risk	FDA Class	MDR Class	Example
Low	Class I	Class I	Bandages
Moderate	Class II	Class IIa	X-Ray-Machines
Moderate to High	Class II/III	Class IIb	Defibrillators
High	Class III	Class III	Pacemakers

**Table 2:** Medical device and automotive standards and guidelines addressing safety and security.

Category	Standard	Standard Title	Description
Development and Life Cycle Processes	IEC 62304	Medical device software—Software life cycle processes	Specification of the software development process for medical device software, and requirements for design, testing, and validation of software
	ISO 24089	Road vehicles—Software update engineering	Design and implementation of processes for global software update standardization.
	ISO 26262	Road vehicles—Functional safety	Guidelines for automotive functional safety addressing systems and components released or under development.
Risk Analysis and Management	EN ISO 14971	Medical devices—Application of risk management to medical devices	Terminology, principles, and the process for risk management of medical devices, including software and the process for recognition of hazards of medical devices
	ISO 24971	Medical devices—Guidance on the application of ISO 14971	Guidance on the development, implementation, and maintenance of a risk management system for medical devices according to ISO 14971
	-	Canadian Premarket Requirements for Medical Device Cybersecurity	Risk analysis and management methods for certain high-risk medical devices.
	SAE J1739	Potential Failure Mode and Effects Analysis (FMEA) Including Design FMEA, Supplemental FMEA-MSR, and Process FMEA	Evaluation of the potential of a failure of a process, a system, and subsystems, services, or designs.
Regulatory requirements and Approval processes	ISO 13485	Medical devices—Quality management systems—Requirements for regulatory purposes	European standard outlining specific requirements for risk management in the life cycle of medical devices
	-	International Medical Device Regulators Forum (IMDRF)	Outlines principles and practices for medical device cybersecurity. Advises use of threat models. Guidelines for efficient realization of regulatory models.
	UN Regulation No. 156	Software update and software update management system	Approval of software updates and SUMS that, among others things, must fulfill safety and security requirements. Processes to protect software updates and verification and validation of functionality and functional safety need to be established by OEMs to be certified.
	UN Regulation No. 155	Cybersecurity and cybersecurity management system	Approval processes of vehicles with regard to cybersecurity and CSMS. OEM is required to set up and implement a management system focusing on cybersecurity over the vehicle life cycle.
Cybersecurity Processes and Management	IEC 81001-1, IEC 81001-5-1	Health software and health IT systems safety, effectiveness and security—Part 5-1: Security—Activities in the product life cycle	Guidelines for the management of cybersecurity in healthcare technology. IEC 81001-1: general introduction to IEC overview of principles, concepts related to cybersecurity in healthcare technology. IEC 81001-5-1: specific guidance on how to manage cybersecurity risks in healthcare technology, structured approach for identifying and evaluating cybersecurity risks, implementing protection measures, and responding to/ recovering from cybersecurity events.
	Article 103 of Regulation (EU) 2017/745	Medical Device Coordination Group (MDCG): Guidance on Cybersecurity for medical devices	Guidelines for medical device manufacturers to fulfill relevant cybersecurity requirements.
	-	FDA Premarket Guidelines: Premarket Submissions for Management of Cybersecurity in Medical Devices	Recognizes need for continuous, iterative approach to device cybersecurity throughout the product life cycle. Provides security risk management strategy and advises manufacturers to be able to identify, assess, and mitigate cybersecurity vulnerabilities. Specifies documentation thereof.
	-	FDA Postmarket Guidelines: Postmarket Management of Cybersecurity in Medical Devices	Specific recommendations managing cybersecurity risks for medical devices in the market: cybersecurity throughout product life cycle: design, development, production, distribution, and deployment maintenance of the device
	SAE J3061	Guidebook For Cyber-Physical Vehicle Systems	High-level guidance on cybersecurity processes, and recommendation for usage of threat analysis and risk assessment methods. Models for discovering threats, assessing the risk of these threats, and analyzing a risk level accordingly.
	ISO/SAE 21434	Road vehicles—Cybersecurity engineering	Definition of an automotive-specific cybersecurity engineering standard concerning the whole vehicle life cycle. Key aspect: TARA for identification of security risks and threats to develop countermeasures and mitigation strategies.
	-	Health Insurance Portability and Accountability Act (HIPAA)	Rules appropriate physical, technical, and administrative safeguards to maintain the confidentiality, integrity, and availability of PHI.

**Table 3:** Risk matrix example based on quantitative probability and qualitative severity [45].

	Negligible	Minor	Serious	Critical	Catastrophic
Frequent	Med.	Med.	Med.	High	High
Probable	Low	Med.	Med.	High	High
Occasional	Low	Low	Med.	Med.	High
Remote	Low	Low	Med.	Med.	High
Improbable	Low	Low	Low	Med.	Med.

**Table 4:** ASIL determination [53].

		Controllability		
Severity	Exposure	C1 >99% Able to Control	C2 >90% Able to Control	C1 <90% Able to Control
S1: Light or Moderate Injury	E1: Very low E2: Low (<1%) E3: Medium (1–10%) E4: High (>10%)	QM QM QM QM	QM QM QM ASIL A	QM QM ASIL A ASIL B
S2: Severe Injury Survival Probable	E1: Very low E2: Low (<1%) E3: Medium (1–10%) E4: High (>10%)	QM QM QM ASIL A	QM QM ASIL A ASIL B	QM ASIL A ASIL B ASIL C
S3: Life Threatening Injury	E1: Very low E2: Low (<1%) E3: Medium (1–10%) E4: High (>ty10%)	QM QM ASIL A ASIL B	QM ASIL A ASIL B ASIL C	ASIL A ASIL B ASIL C ASIL D

**Table 5:** AFR for attack paths of the damage scenario “Incorrect Link and Joint Position Communicated”.

Attack Path ID	Expertise	Knowledge of Item	Window of Opportunity	Equipment	Attack Feasibility Rating
AP1	2	1	0	2	Low (42%)
AP2	2	2	2	3	Med. (75%)
AP3	1	2	2	3	Med. (67%)
AP4	1	2	3	2	Med. (67%)
AP5	2	2	3	3	High (83%)
AP6	2	2	3	3	High (83%)
AP7	1	2	3	2	Med. (67%)
AP8	2	2	3	2	Med. (75%)

**Table 6:** Risk values for threat scenarios in Figure 7.

Threat Scenario	Attack Feasibility	Impact	Risk
	Rating	Rating	Value
Input Signal Spoofed	Medium	Severe	5
Com. GW Tampered	High	Severe	5
SDC Output Signal Spoofed	High	Severe	5

**Table 7:** Simplified SFMEA based on [45].

ID	Item	Function	Failure Mode	Cause of Failure	Potential Effect
R1	Com. GW	Send Joint Positions	Receive Wrong Position	Incorrect Parsing	Collision
R2	Com. GW	Send Joint Positions	Receive Wrong Position	Wrong Joint Reference Position	Collision
R3	Com. GW	Send Joint Positions	Receive Wrong Position	Signal/Service Data Corruption	Collision

## Abbreviations

The following abbreviations are used in this manuscript:

**V2X** Vehicle-to-Everything Communication
**SOME/IP** Scalable service-Oriented MiddlewarE over IP
**OBD** On-Board Diagnostics
**ECU** Electronic Control Unit
**IT** Information Technology
**OEM** Original Equipment Manufacturer
**NIST** National Institute of Standards and Technology
**ASIL** Automotive Safety Integrity Level
**IDS** Intrusion Detection System
**IoT** Internet of Things
**SOA** Service-Oriented Architecture
**CPS** Cyber-Physical System
**IAM** Identity and Access Management
**ADAS** Advanced Driver Assistance Systems
**QM** Quality Management
**OTA** Over The Air
**OR** Operating Room
**HOR** Hybrid Operating Room
**SDC** Service-oriented Device Connectivity
**CPS** Cyber Physical System
**OR table** Operating Room table
**E/E architecture** Electric/Electronic architecture
**HEAVENS** HEAling Vulnerabilities to ENhance Software Security and Safety
**SOTA** Software Over The Air
**HIPAA** Health Insurance Portability and Accountability Act
**TARA** Threat Analysis and Risk Assessment
**FDA** U.S. Food and Drug Administration
**E/E** Electric/Electronic
**FMEA** Failure Mode and Effects Analysis
**SCOT^®^** Smart Cyber Operating Theater^®^
**ICE** Integrated Clinical Environment
**MDPnP** Medical Device Plug and Play
**CSMS** Cybersecurity Management Systems
**AFR** Attack Feasibility Rating
**IMDRF** International Medical Device Regulators Forum
**DFD** Data Flow Diagram
**SUMS** Software Update Management System
**MDR** Medical Device Regulation
**PASTA** Process for Attack Simulation and Threat Analysis
**CAPA** Corrective and Preventive Action
**ICU** Intensive Care Unit
**HIS** Health Information System
**POC** Point of Care
**PHD** Personal Health Device
**NIST** National Institute of Standards and Technology
**BfArM** Federal Institute for Drugs and Medical Devices
**RTOS** Real-Time Operating System
**SFMEA** Software Failure Mode and Effects Analysis
**CRC** Cyclic Redundancy Check
**OCTAVE** Operationally Critical Threat, Asset, and Vulnerability Evaluation
**SAHARA** Security Aware Hazard Analysis and Risk Assessment
**PHI** Protected Health Information
**PnP** Plug and Play
